# Development and Characterization of Biocompatible Cellulose—Tetraphenylethylene Hydrazone Self-Assembling Nanomicelles with Acidity-Triggered Release of Doxorubicin for Cancer Therapy

**DOI:** 10.3390/cimb46120853

**Published:** 2024-12-17

**Authors:** Katia Rupel, Lidia Fanfoni, Jacopo Dus, Martina Tommasini, Davide Porrelli, Barbara Medagli, Federica Canfora, Daniela Adamo, Roberto Di Lenarda, Giulia Ottaviani, Matteo Biasotto

**Affiliations:** 1 Department of Medical, Surgical and Health Sciences, University of Trieste, Strada di Fiume 447, 34149 Trieste, Italy; lidia.fanfoni@gmail.com (L.F.); jacopodus@gmail.com (J.D.); martina.tommasini28@gmail.com (M.T.); bmedagli@units.it (B.M.); rdilenarda@units.it (R.D.L.); gottaviani@units.it (G.O.); m.biasotto@fmc.units.it (M.B.); 2 Department of Life Sciences, University of Trieste, Via Alexander Fleming 31, 34127 Trieste, Italy; dporrelli@units.it; 3 Department of Neuroscience, Reproductive Sciences and Dentistry, University of Naples Federico II, 5 Via Pansini, 80131 Naples, Italy; federica.canfora@unina.it (F.C.); daniela.adamo@unina.it (D.A.)

**Keywords:** cellulose, doxorubicin, drug delivery, nanomicelles, aggregation-induced emission

## Abstract

The development of anticancer diagnostic and therapeutic strategies is of crucial importance to improve efficacy and therapeutic specificity. Here, we describe the synthesis and characterization of fluorescent self-assembling nanomicelles (NMs) based on a biocompatible polysaccharide (cellulose, CE) functionalized with a tetraphenyl ethylene derivative (TPEHy) and loaded with Doxorubicin (DOX) with aggregation-induced emission (AIE) properties and pH-dependent drug release. We obtained CE-TPEHy-NMs with an average diameter of 60 ± 17 nm for unloaded NMs and 86 ± 25 nm for NMs loaded with DOX, respectively. Upon testing different conditions, we obtained an encapsulation efficiency of 86% and a loading capacity of 90%. A controlled dialysis experiment showed that the release of DOX after 48 h is minimal at pH 7.4 (11%), increasing at pH 6.5 (50%) and at its maximum at pH 4.5 (80%). The cytotoxicity of blank and loaded CE-TPEHy-NMs at increasing concentrations and different pH conditions was tested on a MG-63 human osteosarcoma cell line. Based on viability assays at pH 7.4, neither unloaded nor loaded CE-TPEHy-NMs exerted any inhibition on cell proliferation. At pH 6.5, proliferation inhibition significantly increased, confirming the pH-dependent release. We characterized and studied the performance of CE-based amphiphilic, biocompatible NMs for controlled drug release in acidic conditions, such as tumor microenvironments. Further studies are required to optimize their synthesis process and to validate their antitumoral properties in vivo.

## 1. Introduction

The development of new anti-cancer diagnostic and therapeutic strategies is of crucial importance to improving the efficacy and therapeutic specificity of already known and used drugs. Drug delivery systems (DDSs), intended as methods for delivering drugs to their biological site of action and achieving their therapeutic effect using specific carriers, could overcome problems related to conventional chemotherapy, including low specificity, side effects, and cytotoxicity to non-cancer cells, thus improving the pharmacokinetics, bioavailability, biodistribution, and site specificity [1,2].

Nanoparticles (NPs), such as dendrimers, liposomes, micelles, and inorganic materials, have been used widely in antineoplastic therapy as DDSs thanks to the enhanced permeation and retention (EPR) effect, which significantly improves drug penetration and maintenance in tumor cells [3,4]. However, one issue with these nanocarriers is that most antineoplastic drugs are non-emissive; thus, they are not self-traceable, and the majority of DDSs have the sole role of functioning as carriers and forming NPs to deliver the drug into cancer cells [5]. To produce DDSs, amphiphilic polymers have been extensively explored to form nanomicelles (NMs) because of their nanoscale size and their ability to solubilize and deliver hydrophobic pharmaceutical molecules [6]. Notably, NMs designed from natural polymers are attracting much attention due to their high biocompatibility, low immunogenicity, and relatively low cost of production [7]. Cellulose (CE) is a linear-chained polysaccharide composed of β-1,4-D-glucose units with various degrees of polymerization, typically synthesized by a large number of living organisms. CE from diverse origins has been used in drug nanocarriers for in vitro experiments in controlled release systems, showing that they can significantly extend the drug release time due to their morphology, chemical surface reactivity, and physical and biological properties [8,9].

To overcome the limitations of conventional chemotherapy and DDSs, a new class of fluorogens with aggregation-induced emission (AIE) characteristics, AIEgens, has been developed and used to build alternative DDSs [10]. This original class of molecules offers a direct solution to the aggregation-caused quenching (ACQ) phenomenon: the suppression of the light emission of the fluorophore due to π–π stacking, and other non-radiative pathways, correlated to the aggregation of dye molecules with high loading content [11]. Therefore, AIEgens (e.g., in tetraphenylethene, TPE) are almost non-emissive when dissolved in organic solvents, but become highly emissive when spontaneously aggregated at increasing water content, thanks to the restriction intramolecular motion (RIM) effect. Due to these characteristics, these dyes can be chemically bonded to a backbone to form nanoparticles with highly emissive properties that can then be used as DDS containing different anticancer drugs, such as Doxorubicin (DOX) [12,13]. Related to cancer cell therapy, AIEgens were used and incorporated into DDSs in constructs composed, among others, of tetraphenylsilole (TPS) fluorophore and a caspase-3 enzyme specific Asp-Glu-Val-Asp peptide [12], TPE derivative (TPETP) conjugated with a hydrophilic poly(ethylene glycol) chain [14], or TPE with methoxypoly(ethylene glycol) (m-PEG) [13].

Furthermore, one of the challenges in DDS design is to obtain a controlled release of the antineoplastic compound, resulting in higher drug concentrations exclusively in tumor tissue. This can be achieved using various stimuli, such as temperature, redox status, pH, or enzymes [15]. Tumors typically exhibit a slightly acidic microenvironment (ranging from 6.4 to 7.0) compared to normal tissues, caused mainly by the elevated glycolysis rates in cancer cells and hypoxia-induced upregulation of carbonic anhydrase IX [16,17,18,19]. Given this distinct pH difference between healthy and tumor tissues, targeting pH presents a promising strategy for enhancing selective drug release at tumor sites [20].

To the authors’ knowledge, there are a few research groups that have used CE as the hydrophilic core of NMs [9,21,22,23,24,25,26], but none of them employed this polymer to form nanocarriers for DOX with AIE properties. Here, we describe the synthesis and characterization of fluorescent self-assembling NMs using a biocompatible polysaccharide (CE) functionalized with a tetraphenyl ethylene derivative (TPEHy) through an acid-labile hydrazone bond and loaded with DOX with pH-dependent drug release properties in vitro.

## 2. Materials and Methods

### 2.1. Chemicals

Petroleum Ether (EP), Ethyl Acetate (AcOEt), Anhydrous Tetrahydrofuran (THF), Acetonitrile (CH_3_CN), dichloromethane (DCM), methanol (MeOH), ethanol (EtOH), Sodium Chloride (NaCl), Anhydrous Sodium Sulfate (Na_2_SO_4_), Titanium Tetrachloride (TiCl_4_), Potassium Carbonate (K_2_CO_3_), Zinc Metal Powder (Zn), Hydrogen Chloride (HCl), Sodium Hydroxide (NaOH), Trifluoroacetic Acid (TFA), Sodium Metaperiodate (NaIO_4_), Benzophenone (BP), 4-hydroxybenzophenone (BP-OH), bromoethyl acetate (BrEtAc), hydrazine hydrated (NH_2_NH_2_·H_2_O), hydroxylamine hydrochloride (NH_2_OH·HCl), cellulose (CE), Silica Gel for chromatography (SiO_2_), and Doxorubicin (DOX) were purchased from Sigma Aldrich (St Louis, MO, USA). All materials were used as delivered without further purifications.

The 2-(4-(1,2,2-triphenylvinyl)phenoxy)aceto-hydrazide (TPE hydrazide, TPEHy) and di-aldehyde CE (DAC) were synthesized according to protocols previously reported in the literature [27,28,29,30,31].

### 2.2. Synthesis of TPE Derivative NHNH_2_

For the synthesis of TPEHy Derivative 3, 13.40 g of Zn powder (0.205 mol) was placed in a 500 mL three-necked flask, previously dried at 100 °C overnight and saturated with N_2_. Zn powder was suspended in 340 mL of anhydrous THF through constant mixing with a magnetic stirrer. Then, 3 g of BP (0.016 mol) and 4.89 g (0.025 mol) of BP-OH were added to the suspension, and the chemical mixture was left stirring at room temperature for 15 min. The mixture was then cooled in an ice/NaCl bath to −10 °C. Through a dropping funnel, dried and saturated with N_2_, 11.5 mL of TiCl_4_ (0.102 mol) was slowly added, keeping the mixture temperature between −10 and −5 °C. After the addition of TiCl_4_, the mixture was left to heat back to room temperature. The flask was then equipped with a condenser, and the mixture was refluxed (100 °C) for 12 h. After 12 h, the mixture was left to cool to room temperature and then added to a large beaker containing 600 mL of 10% K_2_CO_3_ solution and stirred for 30 min. The solid residue was separated through centrifugation (2500 rpm for 10 min), and the biphasic supernatant was separated through a separating funnel. The reunited organic phases were dried over anhydrous Na_2_SO_4_, filtrated, and evaporated under reduced pressure. The crude reaction product was purified through flash chromatography in a silica gel column using a mixture of EP/AcOEt with 2% gradient of AcOEt from 2–18%. In total, 3 g of TPE-OH was obtained as a pale-yellow solid.

To obtain Derivative 2, in a 50 mL reaction flask, 1.78 g (0.0129 mol) of K_2_CO_3_ was suspended in 34 mL of CH_3_CN. At room temperature, 3 g of Derivative 3 (8.6 mmol) and 1.2 mL (9.5 mmol) of BrEtAc were added in sequence to the suspension. After equipping the flask with a condenser, the mixture was refluxed (100 °C) for 12 h. The mixture was then left cooling back to room temperature and then filtrated under reduced pressure, washing the solid several times with DCM. The reunited organic phases were evaporated, and the crude reaction product was purified through flash chromatography in a silica gel column using a mixture of EP/AcOEt with 1% gradient of AcOEt from 1–12%. In total, 2.5 g of product 2 (5.7 mmol) was obtained as a white solid, with a reaction yield of 37% calculated from BP (2 synthetic steps).

Finally, to synthetize Derivative 1, into a 50 mL flask was placed 2.5 g of Derivative 2 (5.8 mmol) in 28 mL of MeOH. Then, 7 mL of NH_2_NH_2_·H_2_O (0.145 mol) was added to the mixture. The mixture was left stirring at room temperature for 12 h. At the end of the reaction time, water was added to the mixture, and the mixture was left stirring for 15–30 min until a white precipitate completely formed. The precipitate was filtrated under reduced pressure and washed with water. The obtained solid was then dissolved again in DCM and extracted in a separating funnel twice with distilled water and once with a saturated solution of NaCl. The reunited organic phases were dried over anhydrous Na_2_SO_4_, filtrated, and evaporated under reduced pressure, obtaining 2.4 g of product 1 as a white solid (5.7 mmol) with a quantitative yield.

**Figure 1:**
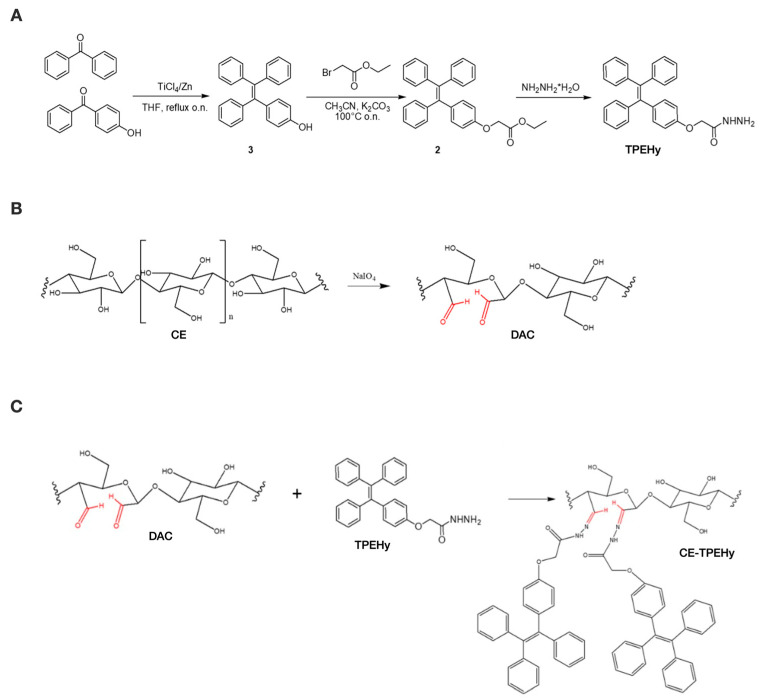
Synthesis phases of the CE-TPEHy polymer. (**A**) Synthesis of 2-(4-(1,2,2-triphenylvinyl)phenoxy)aceto-hydrazide (TPEHy). (**B**) Selective oxidation of cellulose (CE) to di-aldehyde cellulose (DAC). (**C**) Synthesis of CE-TPEHy through the formation of pH-labile hydrazone bond between DAC and TPEHy. Aldehyde groups are highlighted in red.

Structures of TPEHy and intermediates 2 and 3 were validated through NMR spectrometry using a Varian 400 spectrometer operating at frequencies of 400 and 101.56 MHz for ^1^H and ^13^C, respectively. The chemical shifts were calculated using the residual peak of the deuterated solvent as the reference (CDCl_3_ ^1^H 7.26 ppm,^13^C 77.00 ppm; DMSO-d_6_^1^H 2.50 ppm, ^13^C 39.52 ppm). Figure 1A represents the synthesis phases of 2-(4-(1,2,2-triphenylvinyl)phenoxy)aceto-hydrazide (TPEHy). NMR spectra are reported in Supplementary Figure S1.

Using a fluorescence spectrophotometer (Glomax Multi Detection System, Promega Corporation, Madison, WI, USA, ʎ_ex_ = 365 nm; ʎ_em_ = 410–460 nm, UV filter) the ability to self-aggregate of TPEHy was tested by adding increasing % of water (50, 70, and 90% H_2_O (*v*/*v*)) to TPEHy solutions in DMSO. The linearity between the concentration of TPEHy solutions and the intensity of the fluorescence was also tested using 50 to 400 µM solutions of TPEHy in DMSO added with 95% H_2_O (*v*/*v*).

### 2.3. Synthesis of DAC

To obtain DAC, CE was oxidized using methods described in the literature [32,33,34] through NaIO_4_ and KIO_4_ oxidation, as shown in Figure 1B. The effects of different parameters, such as the oxidizing agent, reaction time, temperature, and oxidant concentration, were investigated with the aim of optimizing the reaction. The results showed that NaIO_4_ had the best outcome in forming dialdehyde groups, using a weight ratio of the oxidizing agent NaIO_4_/CE = 1.3 *w*/*w*, a reaction time of 24 h at T = 40 °C, and purification through dialysis against a solution of NaCl 0.2 M for 24 h. In detail, 1 g of CE was placed in a 50 mL Schlenck reactor, sheltered from light exposure by wrapping it with aluminum foil, and suspended in 20 mL of KCl/HCl solution (throughout the reaction, the pH of the suspension was maintained at approximately 1) under constant stirring. Then, 1.3 g of NaIO_4_ (1.3 *w*/*w*) was added to the suspension of CE, and the temperature was increased to 40 °C. After 24 h at 40 °C, 1 mL of glycerol was added and stirred at room temperature for 12 h to eliminate possible residues of the oxidizing agent. The white solid in suspension was then filtrated under reduced pressure and washed with H_2_O and H_2_O/EtOH 1:1 and left drying. The suspension was transferred in a dialytic membrane (cut-off 13,000) and dialyzed against a solution of NaCl 0.2 M for 24 h and then against H_2_O for 2 days. The obtained solid was separated from the majority of H_2_O through centrifugation (3500 rpm, 10 min) and lyophilized.

It was not possible to validate the chemical structure of DAC through NMR analysis due to the solubility issues of the polymer in the solvents commonly used for this type of assay at room temperature (DMSO and H_2_O). Tests using NMR at 80 °C to increase the DAC solubility did not lead to additional information. Thus, for the determination of the percentage of dialdehyde in DAC (DDA%), a modified version of a previously described method consisting of NaOH titration of the HCl developed from the Schiff base formation during the reaction between DAC aldehyde groups and NH_2_OH·HCl was used [29,31,35,36]. Briefly, 50 mg of DAC was suspended in 15 mL of H_2_O, and the pH was adjusted to 4.5 with HCl 0.05 M. Separately, 215 mg of NH_2_OH·HCl was dissolved in 10 mL of H_2_O, and the pH stabilized at 4.5 with NaOH 0.05 M. The solution of NH_2_OH·HCl was added to the DAC suspension and left while constantly stirring in a closed container, at room temperature, for 24 h. Afterwards, the HCl developed was titrated with a solution of NaOH (0.05 M) while monitoring its variation using a pH meter. The percentage of dialdehyde groups (DDA%) in the oxidized polymer can be obtained from the following formula, DA% = (MNaOH·VNaOH)/(mDAC/MwDAC), where MNaOH = molarity of the titrating solution (mol/L); VNaOH = volume of the titrant utilized (L); mDAC = mass of the initial sample (g); and MwDAC = mean molar mass calculated for the unit of DAC (160 g/mol).

### 2.4. Conjugation of the TPEHy Derivative with DAC

The conjugation between TPEHy and DAC was performed through the creation of an acid-sensitive covalent hydrazide bond in DMSO and in the presence of a low quantity of TFA (Figure 1C) to test several conditions, such as the reaction time, temperature, TPE/Polymer equivalent ratio, and type of dialytic solution. The best setting was found using a TPEHy/DAC 5:1 eq ratio for 6 days at T = 40 °C while dialyzing against EtOH to eliminate excess TPEHy. In the final step, the solution was dialyzed (13,000 Da cut-off membrane) and lyophilized. A fluorescence spectrophotometric method (Glomax Multi Detection System, Promega Corporation, Madison, WI, USA) based on a calibration curve (ʎ_ex_ = 365 nm; ʎ_em_ = 410–460 nm, UV filter) was used for the determination of the % of TPEHy conjugated to the oxidized polymer (degree of labeling, DL%). A calibration curve of TPEHy Derivative 1 in DMSO with concentrations ranging from 10 to 500 µM was prepared in a 96-well plate, and then H_2_O was added to each well (95% *v*/*v*). After 1 h, readings determined a linear relationship between the TPEHy concentration and the detected fluorescence in the range 50 to 400 µM (R^2^ = 0.9979). The obtained expression (y = 3.0036 × −113.66) was then used to determine the DL%.

### 2.5. Preparation of CE-TPEHy-NMs

All CE-TPEHy-NMs were prepared from the same stock solution of CE-TPEHy (DDA% = 77%, DL% = 80%) in DMSO at the concentration of 2 mg/mL. NMs were prepared by adding water into aliquots of the solution. The effects of different preparation conditions, such as the time of H_2_O addition (5 min and 36 min dropwise) and the concentration of the starting solution (2 mg/mL, 1 mg/mL, 0.5 mg/mL, 0.25 mg/mL), on the diameter of the final NMs were investigated through DLS analysis.

### 2.6. Characterization and Imaging of CE-TPEHy-NMs

Dynamic Light Scattering (DLS) analysis was performed by employing the Zetasizer Nano ZS with 173° detection optics (Malvern Panalytical, Malvern, UK), and the volume and stability over time of the obtained NMs were evaluated. CE-TPEHy-NMs were visualized through transmission electron microscopy (TEM) and scanning electron microscopy (SEM) at the microscopy center of the University of Trieste. Briefly, 5 µL of the CE-TPEHy-NMs solution was deposited on a carbon grid and dried for 1 h. The grid was then stained with uranyl acetate and imaged with a Philips EM 208 (Philips, Eindhoven, The Netherlands) operating at 100 kV equipped with a Quemesa camera (Olympus Soft Imaging Solutions, Hamburg, Germany) and RADIUS software (Version 2.1, EMSIS, Münster, Germany) for image acquisition and analysis. For SEM analysis, 10 µL of the CE-TPEHy-NMs solution was placed on a glass coverslip mounted on an aluminum stub coated with double-sided carbon tape; the sample was air dried and then coated with a thin layer of gold using an Emitech K550X sputter coater. The images were acquired with a Quanta250 SEM (FEI, Hillsboro, OR, USA) operating in secondary electron mode using an acceleration voltage of 20.00 kV and a working distance (WD) of 9.3 mm.

### 2.7. Loading of CE-TPEHy-NMs with DOX

The ability of CE-TPEHy-NMs to encapsulate drug molecules was tested using the anticancer drug Doxorubicin (DOX) both as hydrochloride salt and in neutral form. Taking advantage of CE-TPEHy’s ability to self-aggregate at increasing water content, DOX-loaded NMs (CE-TPEHy-DOX-NMs) were prepared through the co-loading strategy, which allows for direct encapsulation of the drug during NMs’ aggregation. For determining the CE-TPEHy-NPs’ encapsulation efficiency (EE%) and the loading capacity (LC%), six mixtures of CE-TPEHy and DOX (hydrochloride and neutral) were prepared in DMSO in ratios of 2:1, 5:1, and 10:1. After the self-aggregation mediated by water addition, the excess of DOX in the solution was separated through centrifugation. Encapsulation efficacy (EE%) and loading capacity (LC%) were calculated using Equations (1) and (2), respectively (W = weight).

EE% = W(encapsulated DOX)/W(total DOX)(1)

LC% = W(total DOX)/(W(total DOX) + W(CE-TPEHy nanomicelles))(2)

After preparation, the CE-TPEHy-DOX-NMs were analyzed through DLS and TEM, as described before.

### 2.8. pH-Dependent Doxorubicin Release

The ability of CE-TPEHy-DOX-NMs to release DOX in different pH conditions was tested using a dialysis method. Different conditions were reproduced, with the aim of mimicking several physiological conditions, such as blood and normal tissue (pH 7.4), the extracellular environment of the tumor microenvironment (pH 6.5), and the endosomal environment (pH 4.5) [37,38].

CE-TPEHy-DOX-NMs (EE% = 68%, LC% = 87%) were prepared with DOX hydrochloride in a 10:1 ratio (*w*/*w*). After preparation, CE-TPEHy-DOX-NMs were sealed in separate dialysis bags (3500 Da) and then incubated in 10 mL of solutions at different pH values: PBS pH 7.4, PBS pH 6.5, and acetate/acetic acid buffer pH 4.5. Subsequently, the dialysis bags were placed in an incubator shaker (100 rpm, 37 °C). An aliquot of each dialysis solution (200 µL) was taken at different time points (1 h, 2 h, 3 h, 4 h, 6 h, 12 h, 24 h, 48 h), and the corresponding volume of fresh medium was added to the solutions. The concentration of DOX released was measured using a spectrophotometric method (absorbance at ʎ = 450 nm, Glomax Multi Detection System, Promega Corporation, Madison, WI, USA). The percentage of the drug released was calculated using a calibration curve.

### 2.9. Cell Lines

A human osteosarcoma MG-63 (ATCC number: CRL-1427) cell line was cultured in Dulbecco’s modified Eagle’s medium (DMEM) supplemented with 10% fetal bovine serum (FBS) and 1% penicillin–streptomycin/1% l-glutamine at 37 °C and 5% pCO_2_. Cells were seeded at passage 2–8 (5000 cells/well in 96 multi-well plate) the day before the proliferation assay and incubated in a humidified atmosphere of 5% CO_2_ at 37 °C overnight.

### 2.10. Cell Viability and Proliferation Assays

The growth rate as a function of time of MG-63 cells was assessed by employing the Alamar Blue (Thermo Fisher Scientific, Life Technologies, Waltham, MA, USA) and MTT (Trevigen, Gaithersburg, MD, USA.) viability assays according to manufacturer’s instructions. One day after seeding, cells were incubated with increasing concentrations of blank (unloaded) CE-TPEHy-NMs, loaded CE-TPEHy-DOX-NMs and DOX alone (0.2 μg/mL, 2 μg/mL, and 20 μg/mL). The Alamar blue assay was performed using a pH 7.4 medium, while the MTT assay was performed after the culturing cells in an acidic pH 6.5 medium to evaluate pH-dependent activity. To prepare the acidic medium, 300 μL of a sterile solution of 10% acetic acid was used to reduce the pH of 10 mL DMEM High glucose, 10% FBS, 2 mM Glutamine, 1× Pen/Strep. The pH was controlled both with pH indicator strips and a pH meter. In the Alamar blue assay, the ratio between the fluorescence intensity after 24 hours of treatment and the fluorescence intensity of the same sample at day 0 (seeding time) was reported. For the MTT assay, untreated cells were used to calculate the proportion (%) of proliferating cells in treated wells compared to untreated wells (control) 24 h after treatment. Cells were incubated in a humidified atmosphere of 5% CO_2_ at 37 °C throughout the experimental time. All experiments were performed in biological triplicates.

### 2.11. Statistical Analysis

Origin software (OriginLab Corporation, Northampton, MA, USA) and Prism software (version 9.1.0, GraphPad Software, Inc., 7825 Fay Avenue, Suite 230, La Jolla, CA, USA) were employed for statistical analysis. Data not satisfying normality assumptions (Shapiro–Wilk test) were analyzed by means of non-parametric Kruskal–Wallis and Mann–Whitney tests for comparisons among and within groups, applying Bonferroni’s correction. Two-way ANOVA with post hoc analysis for multiple comparisons was employed to evaluate differences among treatments in the cell proliferation experiment. All statistical assessments were two-sided, and a *p*-value < 0.05 was used for the rejection of the null hypothesis.

## 3. Results

### 3.1. Characterization of CE-TPEHy

The TPEHy derivative and the reaction intermediates (2 and 3) were characterized via NMR. Adding an increasing % of water to DMSO solutions of TPEHy induced self-aggregation and provided an increase in fluorescence, but, notably, the fluorescence of TPEHy in a mixture of 90:10 water:DMSO was 20 times higher than that in a 70:30 mixture. Based on these results, a 95% water mixture was then used for the preparation of the NMs described in the present paper.

Upon analyzing the dependence of the fluorescence on the increase in the TPEHy concentration, the results showed a linear and directly proportional correlation between the tested concentrations (50 to 400 µM).

DAC was obtained in a yield of 65–90%, with a DDA% ranging from 66% to 77%. DAC with a DDA% of 77% was used in the experiment herein described. Conjugation of DAC and TPEHy under different conditions provided a functionalized polymer with a DL% from 45 to 80%. CE-TPEHy conjugate with DL% = 80% was used for NMs’ preparation.

### 3.2. Characterization of Empty and Drug-Loaded CE-TPEHy-NMs

CE-TPEHy nanomicelles (CE-TPEHy-NMs) were prepared using the direct dissolution method. The size of the NMs was highly dependent on both investigated parameters: the timing of water addition and the CE-TPEHy concentration. DLS analysis showed an increase in the NMs’ size by decreasing the time of water addition and a decrease in the NMs’ size by decreasing the concentration of the starting solution. In particular, the 5 min dropwise addition of 5.5 mL of water to 1 mL of 0.25 mg/mL CE-TPEHy solution in DMSO, followed by dialysis against H_2_O, provided empty auto-aggregating fluorescent CE-TPEHy-NMs with a desirable diameter ranging from 60 ± 17 nm. The stability of the CE-TPEHy aggregation process was tested through DLS by analyzing aqueous suspensions of NPs stored for up to one month at 4 °C, which found no variations in their size and scattering.

**Figure 2:**
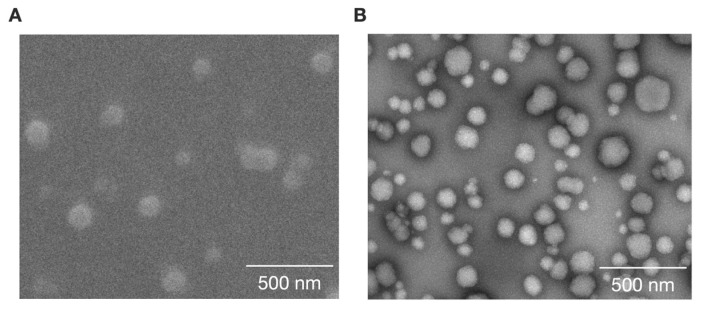
Characteristics of loaded and unloaded CE-TPEHy-NMs. (**A**) Representative SEM image of unloaded CE-TPEHy-NMs. (**B**) Representative TEM image of loaded CE-TPEHy-DOX-NMs.

The DOX-loaded NMs (CE-TPEHy-DOX-NMs) were prepared using the “co-loading” strategy through dropwise addition of 5.5 mL of water to 1 mL of DOX/CE-TPEHy solution in DMSO. The acquired TEM and SEM images confirmed the presence of spherical NMs with diameters compatible with what was detected upon DLS analysis, ranging from 86 to 25 nm. Figure 2A,B show representative SEM and TEM images.

### 3.3. Drug Encapsulation Efficiency and Loading Capacity of CE-TPEHy-NMs

In the case of using DOX hydrochloride, the EE% ranged from 26% to 68% and the LC% from 34% to 87% (with DOX/CE-TPEHy ratios of 2:1, 5:1, and 10:1, respectively). In contrast, using neutralized DOX, regardless of the DOX/CE-TPEHy ratio used, the EE% was about 70% in all cases, and the LC% ranged from 58% to 90% as the concentration of neutralized DOXO increased. The best combination of EE% and LC% was obtained by using a 10:1 DOX/CE-TPEHy ratio with both neutralized and hydrochloride DOX (DOX:CE-TPHy *w*/*w*). The values of EE% and LC% are shown in Table 1.

### 3.4. pH-Dependent Doxorubicin Release

**Figure 3:**
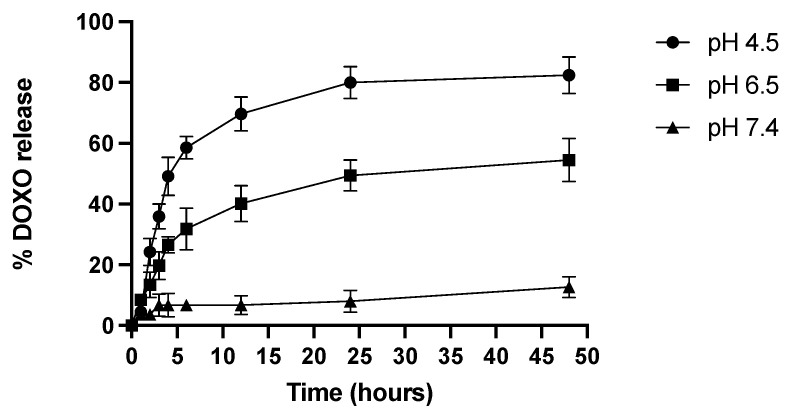
DOX release from CE-TPEHy-DOX-NMs at different pHs.

The DOX release from CE-TPEHy-DOX-NMs was evaluated using a classic dialysis method. Compared with normal tissue, the tumor microenvironment is usually weakly acidic [20]. Herein, three different mediums were tested with increasing acidity (pH 7.4, 6.5, and 4.5). The results confirmed the pH-dependent drug release profile, made possible thanks to the acid-labile hydrazone bond, as represented in Figure 3. The results showed that at pH 7.4, the release is minimal (6.7% at 4 h, 7.9% at 24 h, and 12.7% at 48 h), and at pH 6.5, it reaches 26.6% at 4 h, 49.3% at 24 h, and 54.5% at 48 h. Meanwhile, at pH 4.5, the release is at its maximum (49.13% at 4 h, 79.9% at 24 h, and 82.5% at 48 h).

### 3.5. pH-Dependent Inhibitory Effect of Loaded and Unloaded CE-TPEHy-NMs on Tumor Cells’ Viability and Proliferation

**Figure 4:**
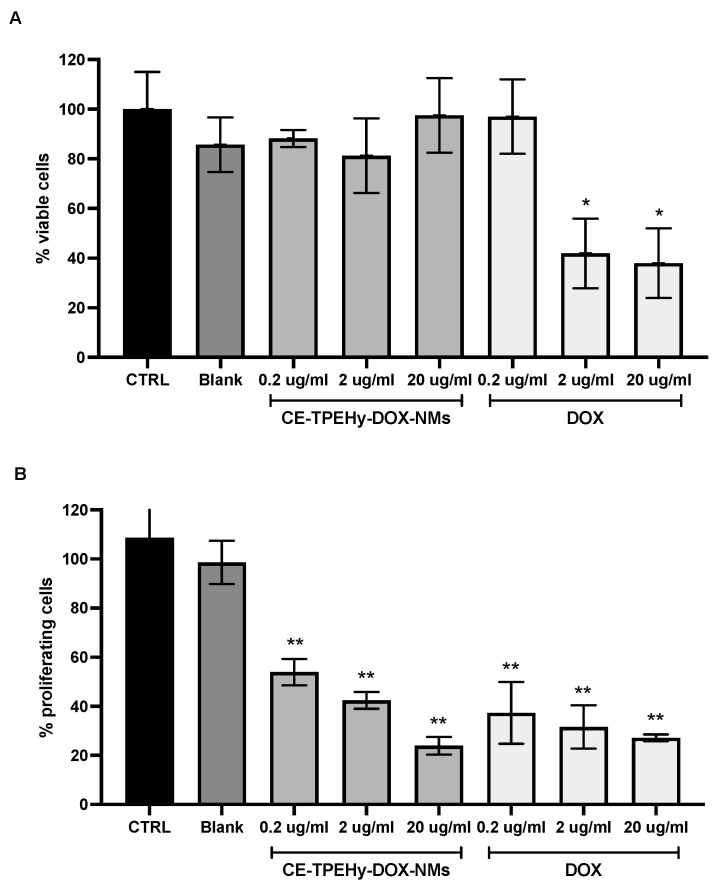
Cell viability and proliferation assays at different pHs on human osteosarcoma MG-63 cells treated for 24 hours. (**A**) Alamar blue assay performed at pH 7.4. *CTRL* untreated cells. *Blank* unloaded CE-TPEHy-NMs. Concentrations refer to DOX content. * Mann–Whitney U-test *p* < 0.05 treated versus control. (**B**) MTT assay performed at pH 6.5. *CTRL* untreated cells. *Blank* unloaded CE-TPEHy-NMs. Concentrations refer to DOX content. ** Two-way ANOVA *p* < 0.001 treated versus control.

The possible inhibitory efficacy of unloaded CE-TPHy-NMs and loaded CE-TPHy-DOX-NMs was evaluated on tumor osteosarcoma cells MG-63. Unloaded CE-TPEHy NMs did not exert any inhibitory effect on cells for every concentration tested (Mann–Whitney U-test *p* = NS), thus confirming their biocompatibility. DOX alone was effective at significantly inhibiting cell proliferation at concentrations of 2 μg/mL and 20 μg/mL (Mann–Whitney U-test *p* < 0.05 for both) but not at 0.2 μg/mL (Mann–Whitney U-test *p* = NS). When DOX was delivered encapsulated in CE-TPEHy-DOX-NMs, it did not show any significant inhibition of cell proliferation for all concentrations tested (0.2 μg/mL, 2 μg/mL, and 20 μg/mL (Mann–Whitney U-test *p* = NS)), which is consistent with the fact that at pH 7.4, the release of DOX from NMs is minimal (7.9%), as shown in the pH-dependent release experiment. The results of the cell viability Alamar blue assay are represented in Figure 4A.

The effect of CE-TPHy-DOX-NMs on tumor osteosarcoma cells’ proliferation activity was then tested at pH 6.5 at different concentrations to mimic the tumor’s acidic environment. Unloaded CE-TPEHy NMs did not exert any inhibitory effect on cells for every concentration tested (two-way ANOVA *p* = NS), also confirming biocompatibility in this setting. DOX alone was effective at significantly inhibiting cell proliferation for all concentrations tested (0.2 μg/mL (two-way ANOVA *p* < 0.001), 2 μg/mL (two-way ANOVA *p* < 0.001), and 20 μg/mL (two-way ANOVA *p* < 0.001)). Also, when DOX was delivered encapsulated in CE-TPEHy-DOX-NMs, it showed significant inhibition for all concentrations tested (0.2 μg/mL (two-way ANOVA *p* < 0.001), 2 μg/mL (two-way ANOVA *p* < 0.001), and 20 μg/mL (two-way ANOVA *p* < 0.001)). The results did not show any significant difference between DOX alone and loaded in NMs, as represented in Figure 4B. These results are consistent with the different release rates of the encapsulated drug at diverse pH values: at pH 7.4, mimicking physiological conditions of healthy tissues and blood streams, the release rate was minimal after 24 hours (7.9%), while in a setting of acidic pH, such as tumor microenvironments (pH 6.5), the release rate of DOX was 54.5% at the same time point.

## 4. Discussion

The present work aimed to describe the synthesis and characterization of fluorescent self-assembling NMs using a biocompatible polysaccharide (CE) functionalized with a tetraphenyl ethylene derivative (TPEHy) through an acid-labile hydrazone bond and loaded with a drug used in conventional chemotherapy (DOX), with AIE properties and pH-dependent drug release in vitro.

Among several type of AIEgens, we chose TPE derivatives as chemical components of fluorescence sensors due to their self-aggregation properties, high photostability, simple synthesis, and easy structural modification [39,40]. As the backbone of the herein described DDS (CE-TPEHy-NMs), the cheap, biocompatible, easily accessible polymer CE was chosen. Other authors have described the synthesis of NMs with CE as the hydrophilic core and different hydrophobic compounds. CE was conjugated with polyethylene glycol [9,25], JEFFAMINE [26], poly (p-dioxanone) [24], linoleic acid [23], poly(e-coprolactone) [21], and poly(lactide) [22]. The mean diameters of these NMs were variable, from 20–50 nm to 162–340 nm, depending on the aggregation conditions and the loaded content. Regarding the encapsulation conditions, throughout the studies and consistently in our results, a ratio between NMs and the drug of 1:10 seemed to yield the best loading capacity and encapsulation efficiency values. The size of NMs is one of the critical factors for obtaining the EPR effect, which is a significant aspect of delivering anticancer agents efficiently to targeted tumors. In fact, tumor vessels are highly permeable to macromolecular compounds, which remain trapped inside of the tumor tissue for a prolonged period due to dysfunctional lymphatic drainage [41,42]. Studies have shown that EPR is most effective with nanoparticles in the size range of 20–200 nm, while smaller compounds diffuse back to the circulation [43]. We obtained an average diameter of 60 ± 17 nm for unloaded NMs and 86 ± 25 nm for NMs loaded with DOX, as confirmed via DLS and both SEM and TEM imaging, making CE-TPEHy-NMs suitable for accumulating in the intratumoral interstitial spaces.

The synthetic pathway involved the selective biopolymer’s oxidation and subsequent conjugation with TPEHy through a pH-labile hydrazone bond that, being susceptible to hydrolysis at acidic lysosomal pH, allows for the intracellular release of the anticancer drug from the NMs. Hydrazone bonds have been used before in DDSs [44,45] thanks to their ease of synthesis, modularity, and stability towards hydrolytic cleavage [46]. While their synthesis can be accomplished through three major synthetic pathways, (a) condensation between hydrazines and ketones or aldehydes, (b) Japp–Klingemann reaction in which aryl diazonium salt and beta-keto ester or acid coupling leads to the formation of hydrazones, and (c) reaction between aryl halides and non-substituted hydrazones [46], in most of the described conjugates, including our polymer, the first method is employed [47].

We demonstrated that CE-TPEHy-NMs load the drug inside of the micelle and remain stable in the neutral pH environment, e.g., the blood stream, but selectively release the loaded drug through dissociation of the hydrazone bond in an acidic context, such as the tumor microenvironment (with a pH ranging from 6.4 to 7.0). After 48 h, the release is minimal at pH 7.4 (mean 13%), increasing at pH 6.5 (mean 55%) and at its maximum at pH 4.5 (83%). In a previous study describing an amphiphilic copolymer (H40-P(LA-DOX)-b-PEG-OH/FA) conjugated with DOX through a pH-sensitive hydrazone bond, DOX release after 45 h was found to be 15%, 90%, and 83% at pH 7.4, 5.3, and 6.6, respectively [48]. Other compounds, including hydrazone bonds forming NMs for drug delivery, have been described. For instance, Liu et al. described a multifunctional polymer oligomeric hyaluronic acid–hydrazone bond folic acid–biotin that proved to be pH-sensitive forming nano-actiniaes loaded with icariin and curcumin, which showed drug release after 48 h of approximately 15% at pH 7.4, 45% at pH 6.8, and 70% at pH 4.8 [49]. Xiong et al. showed that about 90%, 30%, and 10% of DOX was released from the pH-sensitive prodrug nanomicelles obtained through the coupling of DOX and vitamin E succinate via a hydrazone bond in 72 h at pH 5.0, 6.4, and 7.4, respectively [50]. Liang et al. evaluated the pH-driven drug release from pluronic-docetaxel micelles containing a hydrazone bond, showing a cumulative release amount of docetaxel of ~84.9%, which was six times higher than that at pH 7.4 [51]. Thus, the release rates described in our study are consistent with data reported with previous studies.

Other strategies to obtain pH-dependent drug release NMs containing CE have been described, such as ester bonds. Singam et al. synthetized PEGylated ethyl CE self-assembling micelles with a diameter of blank micelles of approximately 80 nm and loaded ranging from 154 to 250 nm, with a loading efficiency of 33–53%. In vitro, drug release studies were performed for 48 h, where 100% DOX release was recorded at pH 5.5 and 52% DOX release was recorded at pH 7.4 from the micelles [9]. Compared to our data, we obtained a system that has lower release rates at physiological pH. This is crucial for a DDS that aims at causing low toxicity, avoiding the release of antineoplastic toxic drugs in neutral compartments, and potentially limiting side effects for healthy tissues.

The AIE phenomenon is widely used to construct different types of probes for chemosensors and bioimaging to be used both in vitro and in vivo [10]. Among others, TPE is a commonly used AIE molecule, with a major limitation being its scarce solubility in aqueous mediums [52], requiring its conjugation to water-soluble compounds or modification with the introduction of hydrophilic groups, such as hydrazone groups. Previously published studies have successfully synthetized CE-based polymers, including TPE derivatives with AIE properties, to be used as chemosensors. For instance, Wang et al. described a polymer synthetized from methylcellulose (a water-soluble derivative of CE) and TPE via azide–alkyne click reaction, which showed self-assembling properties into micelles with a mean diameter of 42 ± 6 nm in water [53]. Moreover, the same author reported the synthesis of an aggregation-induced emission (AIE) sensor for the detection of Fe^3+^ ions, which was fabricated through the electrostatic interaction between 1,1,2-triphenyl-2-[4-(3-sulfonatopropoxyl)-phenyl]-ethene sodium salt (SPOTPE) and quaternized CE (QC) [54].

To the authors’ knowledge, this is the first description of CE and a TPEHy-based biocompatible polymer forming self-assembling NMs suitable for drug delivery of hydrophobic drugs designed including a pH-labile hydrazone bond, where the covalent conjugation between DAC and TPEHy happens through the reaction of aldehydic and hydrazine groups. The chemical nature of the hydrazone bond that allows for pH-dependent drug release is an advantage for selective action in acidic environments, such as solid tumors, while avoiding drug release in other physiological liquids, such as the blood stream or interstitial liquid. The next phases will include the evaluation of the ability of CE-TPEHy-DOX-NMs to be internalized into cancer cells in vivo and exert their inhibitory activity. Furthermore, the pathway of internalization will be investigated. Indeed, it has been shown that nanoparticles with positive charge tend to interact with the negatively charged proteins at the cell surface to form clathrin vesicles, which then fuse with endosomes/lysosomes where the acidic environment promotes the cleavage of chemical bonds, leading to the rapid release of the loaded molecule [55]. Finally, the next step will be the validation of their antitumoral properties in vivo to confirm their potential as DDSs in cancer therapy and to utilize this technique as tool to study drug delivery and distribution in the tumor mass of xenograft murine models.

## 5. Conclusions

We synthetized and characterized an amphiphilic, biocompatible biopolymer using oxidized CE and a tetraphenyl ethylene derivative that has AIE and self-assembling properties in NMs in an aqueous environment, with an acid-sensitive hydrazone bond for controlled drug release in acidic conditions, such as tumor microenvironments. The sizes of the DOX-loaded NMs were larger (86 ± 25 nm) than the size of the blank NMs (60 ± 17 nm), as confirmed using DLS, SEM, and TEM imaging, with a diameter suitable for obtaining the EPR effect. The DOX-loaded NMs demonstrated major and faster release of the drug in acidic environments (pH of 4.5 and 6.5), whereas slower and minimal DOX release was observed at a physiological pH of 7.4. Furthermore, cell viability assays on osteosarcoma MG-63 cells performed at pH 7.4 showed that neither unloaded nor loaded CE-TPEHy-NMs exerted any inhibition of cell proliferation. At pH 6.5, proliferation inhibition significantly increased, confirming the pH-dependent release. Further studies are required to validate their antitumoral properties in vivo and to confirm their potential as DDSs in cancer therapy.

## Figures and Tables

**Table 1:** Encapsulation efficiency (EE%) and loading capacity (LC%) of nanomicelles. Different conditions according to the reagent weight/weight ratio were tested. ^a^ neutralized DOX.

DOX/CE-TPHy *w*/*w*	EE%	LC%
2:1	26	34
2 ^a^:1	70	58
5:1	41	67
5 ^a^:1	69	77
10:1	68	87
10 ^a^:1	86	90

## Data Availability

All data can be requested upon reasonable request to the corresponding author.

## Supplementary Materials

The following supporting information can be downloaded at https://www.mdpi.com/article/10.3390/cimb46120853/s1, Figure S1. The ^1^H NMR spectra recorded using a Varian 400 spectrometer operating at a frequency of 400 MHz. (A) TPEHy Derivative 3. (B) TPEHy Derivative 2. (C) TPEHy Derivative 1.
