# Supplementary figures and images for: Development and Characterization of Biocompatible Cellulose—Tetraphenylethylene Hydrazone Self-Assembling Nanomicelles with Acidity-Triggered Release of Doxorubicin for Cancer Therapy
Source: Curr Issues Mol Biol. 2024 Dec 17;46(12):14244–58. doi: 10.3390/cimb46120853 (PMC11674980; doi:10.3390/cimb46120853)

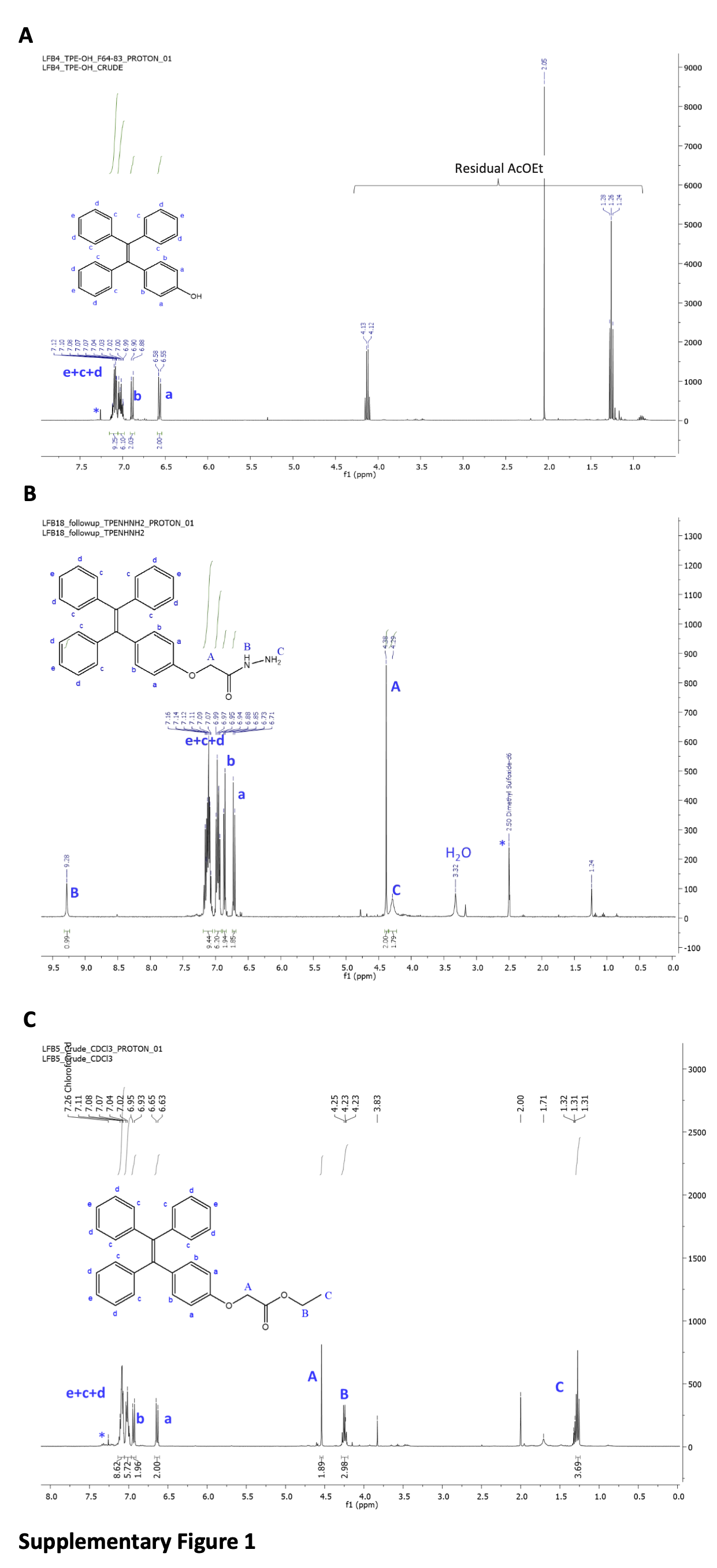

Supplement: Supplementary file 1 [file cimb-46-00853-s001.zip › cimb-3341127-supplementary.png]
